# A randomised controlled feasibility trial of an early years language development intervention: results of the ‘outcomes of Talking Together evaluation and results’ (oTTer) project

**DOI:** 10.1186/s40814-023-01333-y

**Published:** 2023-06-29

**Authors:** Dea Nielsen, Katrina d’Apice, Rachael W. Cheung, Maria Bryant, Rebecca Heald, Chloe Storr, Louise Tracey, Rukhsana Rashid, Josie Dickerson, Claudine Bowyer-Crane

**Affiliations:** 1 Department of Health Sciences, University of York, York, UK; 2 Population Health Sciences, Bristol Medical School, University of Bristol, Bristol, UK; 3 Department of Health Sciences/Hull York Medical School, University of York, York, UK; 4 BHT Early Education and Training, Bradford, UK; 5 Bradford Institute of Health Research, Bradford, UK; 6 National Institute of Economic and Social Research, London, UK

**Keywords:** Early language intervention, Home learning environment, Feasibility study, Early language delay

## Abstract

**Background:**

Early language difficulties are associated with poor school readiness and can impact lifelong attainment. The quality of the early home language environment is linked to language outcomes. However, few home-based language interventions have sufficient evidence of effectiveness in improving preschool children’s language abilities. This study reports the first stage in the evaluation of a theory-based programme, Talking Together (developed and delivered by BHT Early Education and Training) given over 6 weeks to families in the home setting. We aimed to test the feasibility and acceptability of delivering Talking Together in the Better Start Bradford community prior to a definitive trial, using a two-armed randomised controlled feasibility study.

**Methods:**

Families from a single site within the Better Start Bradford reach area were randomly allocated (1:1) to the Talking Together intervention or a wait list control group. Child language and parent-level outcome measures were administered before randomisation (baseline), pre-intervention (pre-test), 2 months post-intervention start (post-test), and 6 months post-intervention start (follow-up). Routine monitoring data from families and practitioners were also collected for eligibility, consent, protocol adherence, and attrition rates. Descriptive statistics on the feasibility and reliability of potential outcome measures were analysed alongside qualitative feedback on trial design acceptability. Pre-defined progression-to-trial criteria using a traffic light system were assessed using routine monitoring data.

**Results:**

Two-hundred and twenty-two families were assessed for eligibility; of these, 164 were eligible. A total of 102 families consented and were randomised (intervention: 52, waitlist control: 50); 68% of families completed outcome measures at 6-month follow-up. Recruitment (eligibility and consent) reached ‘green’ progression criteria; however, adherence reached ‘amber’ and attrition reached ‘red’ criteria. Child- and parent-level data were successfully measured, and the Oxford-CDI was identified as a suitable primary outcome measure for a definitive trial. Qualitative data not only indicated that the procedures were largely acceptable to practitioners and families but also identified areas for improvement in adherence and attrition rates.

**Conclusions:**

Referral rates indicate that Talking Together is a much-needed service and was positively received by the community. A full trial is feasible with adaptations to improve adherence and reduce attrition.

**Trial registration:**

ISRCTN registry ISRCTN13251954. Retrospectively registered 21 February 2019

**Supplementary Information:**

The online version contains supplementary material available at 10.1186/s40814-023-01333-y.

## Key messages regarding feasibility

- What uncertainties existed regarding the feasibility? The key uncertainties for evaluation were the acceptability of a waiting control evaluation design and retention to the intervention. The intervention was targeted towards children identified as in need of additional support, and it was unclear whether families would agree to take part in a trial, as this would mean a 50% chance of waiting 6 months before receiving treatment. Retention was a key issue, as the Talking Together programme has a 27% attrition rate. Finally, we needed to establish the acceptability of data collection and measures for both families and practitioners, as additional measures had been added as part of the service design process.
- What are the key feasibility findings? The study showed that we were able to recruit participants to the trial, although attrition and adherence rates did not meet green progression criteria. Procedures and measures were largely acceptable for both practitioners and families. However, additional training for language development workers (LDWs) is needed to ensure all eligible families are offered the chance to participate in a trial.
- What are the implications of the feasibility findings for the design of the main study? The results indicate that it is feasible to carry out a randomised design to test the effectiveness of the Talking Together programme, although procedural changes need to be made to address attrition and adherence. Qualitative feedback identified where trial strategies could be improved, for example, increased training to alleviate practitioner concerns about the waiting control group and ensure that the intervention is offered to all eligible families, and providing key information to families in different ways to improve participants’ understanding of the trial during the consent process.

## Background

A concerning number of UK children start school with language skills below what is expected for their age [1, 2], with children from deprived areas showing a higher incidence of language difficulties compared with their more affluent counterparts [3]. Reports indicate that between 5 and 8% of children across the UK have been shown to experience language difficulties, with this figure rising to 20% in areas of deprivation [4]. Importantly, evidence is emerging that COVID-19 may have increased the number of young children with language and communication delay [5] and reports suggest that fewer children are ‘school ready’ than before the pandemic [6]. As children’s language ability is associated with their school readiness and long-term academic outcomes [7], children’s language development in deprived communities must be supported in order to reduce the attainment gap.

Previous studies have identified the home learning environment as pivotal in developing young children’s language skills [8]. Whilst the importance of early identification and intervention in the face of potential language delay has been emphasised, there are few evidence-based interventions that have been proven to improve children’s language abilities [9]. The Talking Together intervention was commissioned to support disadvantaged families through the Better Start Bradford programme (https://www.betterstartbradford.org.uk/). Talking Together is a theory-based programme designed and delivered in the home by the registered charity BHT Early Education and Training. The programme aims to equip parents with the knowledge, skills and confidence to effectively engage with their child and thus improve the home learning environment, which in turn is expected to enhance children’s language skills [7, 10]. The Talking Together programme has been running in the community in Bradford since 2006 and as a part of the Better Start Bradford programme since 2016. Although theory-based, the effectiveness of Talking Together to improve children’s language skills is currently not known. The objective of the current study, ‘oTTer: outcomes of Talking Together evaluation and results’, was to undertake a feasibility randomised controlled trial of the Talking Together intervention to decide whether a full-scale randomised controlled trial of the intervention would be possible and justified.

## Methods

### Aims

The oTTer study had two aims: (1) to assess the feasibility and acceptability of conducting a definitive randomised controlled trial to evaluate the effectiveness of the Talking Together intervention and (2) to identify challenges with the implementation and delivery of the Talking Together programme as part of a trial.

## Research objectives

The following objectives were identified for aim 1:

1. To assess the acceptability of a waiting list control method, including the impact on recruitment and attrition rates
2. To assess whether those who take part in the feasibility trial are representative of the wider population who receive Talking Together as an intervention
3. To assess the reliability and responsiveness of potential outcome measures for inclusion in a future definitive trial
4. To provide an estimation of sample size based on intervention completion, attrition and responsiveness of outcome data to allow planning for a randomised controlled trial
5. To determine the acceptability of trial procedures, including data collection and outcome measures

The following objectives were identified for aim 2:

1. To track protocol adherence rates and quality assurance associated with implementation and delivery of Talking Together as part of a trial, as well as any moderators of challenges faced
2. To identify the time and resources required to train practitioners to administer the intervention, and how these relate to resource requirements for definitive RCT development

### Design

The oTTer study was a single site, randomised controlled study comprising an intervention arm and a waiting control arm. Allocation took place at the individual level using a 1:1 ratio. The methodology is outlined below, and full details are available in the published protocol [11]. The protocol was adhered to with a few exceptions: (a) fidelity data was not saved by the service provider, so it was not possible to use this data in our analyses; (b) the randomisation programme was amended following an allocation violation to avoid reoccurrence and (c) the original progression criteria were changed for clarity (http://www.isrctn.com/ISRCTN13251954).

### Setting

The oTTer study was conducted in the Better Start Bradford area which covers three ethnically diverse and deprived inner-city areas of Bradford, a city in North England, making up approximately 12% of the Bradford region [12]. Prior to COVID-19, approximately 24% [13] of children in  the Born in Bradford's Better Start (BiBBS) cohort were identified as late talkers.

### Intervention (Talking Together)

The Talking Together programme comprises (a) a Universal Language Assessment (ULA) offered to all 2-year-olds in the Better Start Bradford area and (b) the Talking Together intervention offered to those children identified as at risk of language delay on the basis of the ULA. The local Health Visiting Service provide BHT with the details of all children turning 2 years old in the following month within the Better Start Bradford region. Letters are then sent offering all families the BHT ULA. This assessment focuses on children’s primary language skills and is delivered in the home, in the predominant language of the family (whenever possible).

The Talking Together intervention aims to equip parents with a greater understanding of children’s language development and to improve parent–child interactions and the home learning environment. The intervention is appropriate for use with both monolingual and multilingual children, regardless of whether English is one of the target child’s languages. This is because the intervention focuses on developing parents’ skills to provide a stimulating language environment in the home, and the behaviours the intervention targets are not language specific. The ULA that is used to refer children to the intervention can be used with both monolingual and bilingual children because it specifically requests that parents respond based on all their child’s languages. As such, whilst multilingual children’s language skills and vocabulary may be distributed across multiple languages (i.e. they may know a word in only one of their languages), their language skills in any language are reflected in the assessment. This ensures that children who are non-English speaking monolinguals or multilinguals are not disadvantaged or disproportionally referred to the service.

Talking Together is delivered by BHT language development workers (LDWs), who have undergone 31.5 full days of training that includes ICAN Early Talk [14], Elkan [15], Makaton [16], safeguarding, data protection and the Bradford protocol for missing children [17]. Trainees also shadow a LDW delivering the full intervention, deliver one complete intervention alongside a qualified LDW themselves, and deliver another with observation and feedback.

The Talking Together programme encompasses weekly, home-based sessions for 6 weeks, totaling six individual sessions, delivered 1:1 with parents and children. Each session focuses on a different topic area, including *what is communication*, *the importance of play*, *attention and listening*, *turn-taking*, and *praise and encouragement*. The final overview session is a plenary covering the content in the preceding five sessions. The first and last sessions also include parent and child outcome measures.

### Waiting control

Families in the waiting control group received a pack containing information on children’s language development, signposting to additional online resources, ideas and materials for activities to support early communication and a story book, but no guided input.

### Access to other services

As families were part of the Better Start Bradford reach area, all families were also eligible to access other interventions provided by Better Start Bradford (https://www.betterstartbradford.org.uk), including interventions for children’s language, socioemotional development, and nutrition. They would also be eligible for other services provided in the district. However, no routine or voluntary services for children in this age group closely mirrored the contents of the Talking Together intervention. Furthermore, as this intervention considers the home visiting model central to its appeal, it is particularly relevant that there were no other home-visiting interventions focused on supporting children’s language and communication available at the time of implementation. This suggests there was limited risk of parents with concerns for their child’s language development accessing other services, particularly given long waiting lists for speech and language services [18].

### Eligibility criteria

Families were invited to participate in the oTTer study if they met the following inclusion criteria:

- they had been referred to Talking Together by a LDW following the ULA
- they consented to receive the intervention in their home
- they lived within the catchment area of Better Start Bradford
- the target child was aged between 2 to 2.5 years at the time of recruitment
- they nominated a specific family member to receive the entirety of the intervention
- they consented to randomisation and accepted that if they were allocated to the control group, they would wait 6 months to receive the intervention and would be visited for additional data collection during the wait
- they spoke primarily English, Urdu, or Punjabi with the target child. This eligibility criteria was due to the importance of assessing children in their native language, which was only possible for languages spoken by both the LDWs and research assistants

Families were excluded from the study if they met any of the following criteria:

- the target child had a known sensory impairment or developmental disorder
- their referral into Talking Together came from a source other than a LDW (i.e. safeguarding authorities)
- they were unable to confirm a specific family member to participate in the entirety of the intervention

### Recruitment and consent

Following the ULA, families referred to Talking Together and who met oTTer eligibility criteria were offered the opportunity to take part in the trial. They were provided with an information sheet and consent form which were explained by the LDW. Parents were told that they were eligible to be part of the trial, but they did not have to participate to receive the intervention. It was also made clear that being part of the trial would mean they either received the intervention immediately or after a 6-month interval, whilst if they did not participate in the trial, they would be put on the standard intervention waiting list (usually less than 6 months). No other incentives were offered for participation in the trial. Informed consent was taken from families by the LDWs. Those who did not consent to be part of oTTer were added to the standard waiting list for the Talking Together intervention outside of the trial. Our target sample size for the trial was 120 participants, with a minimum sample set at 60 participants (30 per group) in line with the literature [19, 20].

Consent for qualitative interviews from families who were part of oTTer was taken at the point of invitation by LDWs during assessment visits, where it was made clear they would be contacted by a research assistant to arrange the interview. Families had the opportunity to withdraw consent from the trial at any point.

### Randomisation and blinding

Families who consented to take part in the oTTer study were randomised to either the intervention or waiting control group on a 1:1 basis, using minimisation to control for (a) language of delivery (English, Urdu, or Punjabi), (b) whether more than one child would be present during delivery, and (c) Children’s Centre reach area. Data collection occurred simultaneously across the two treatment arms, by LDWs in the intervention group and research assistants in the waiting control group. As a result, it was not possible to blind the research team to treatment allocation at pre- and post- test. Follow-up data collection was carried out by someone who had not previously worked with the child (research assistants for the intervention arm, LDWs for the waiting control arm).

### Data collection

**Fig. 1:**
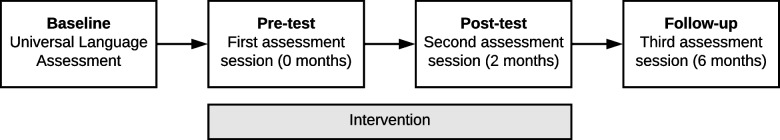
Time points and timescale of data collection

There were four time points in the oTTer trial: baseline, pre-test, post-test at 2 months after the intervention started, and follow-up at 6 months after the intervention started (Fig. 1). Routine monitoring data, such as the number of families in the trial, was collected at each time point (see protocol for details [11]). At baseline, data from the ULA measures was also collected. At all intervention time points (pre-test, post-test, and follow-up), potential primary and secondary outcome data were collected (Fig. 1). Qualitative data regarding the process evaluation was collected from staff after recruitment and at the end of the follow-up period for families.

#### Identification of appropriate outcome measures

We tested several screening, primary, and secondary outcome measures for potential use in a definitive trial, and the full details of all measures can be found in the published protocol [11].

The ULA comprised two measures:

1. BHT Language Screener: this measure was created by BHT in conjunction with speech and language therapists as well as academic partners and comprises ten statements about children’s current language skills scored on a 3-point scale of frequency occurrence (the child ‘does not do this yet’, ‘does this sometimes’, ‘does this often’). The specific reason for the referral into the programme is also noted.
2. The Oxford Communication Development Inventory-Short (CDI-Short) [21]: a parent-reported checklist of 100 vocabulary items that the child can (a) say and (b) understand.

Two measures were considered for suitability as the primary outcome for a future trial:

1. The Oxford Communication Development Inventory-Short (CDI-Short) [21]: Following screening, this assessment is re-administered as an outcome measure.
2. The WellComm language assessment [22]: an assessment of children’s language development conducted by LDWs. This measure was administered by LDWs and research assistants in the child’s native language, but language skills were assessed holistically, so they received credit regardless of the language of their response. The measure has been used previously in English, Urdu, and Punjabi.

In addition, we evaluated three measures for feasibility as secondary outcomes:

1. Maternal Object Relations Scale (MORS) [23]: a parent-reported measure assessing parent/carer and child relationships and attachment.
2. Home Learning Environment Questionnaire (HLEQ) [24]: a parent-reported indicator of the types and frequency of activities in the home shown to be predictive of children’s later language skills.
3. Strengths and Difficulties Questionnaire (SDQ): Hyperactivity and conduct subscales [25]: a parent-reported measure of children’s emotional and behavioural adjustment.

#### Quantitative data analysis

Statistical analyses were conducted to explore the appropriateness of the outcome measures in terms of their reliability and responsiveness. Cronbach’s alpha (*α*), a measure of internal consistency and reliability, was computed for each outcome measure in R (version R 4.0.2) based on available item-level data at baseline (ULA) and pre-test (HLE, MORS, SDQ). Cronbach’s alpha for the Oxford-CDI short and the WellComm could not be calculated, as item-level scores were not available for these measures.

The responsiveness of each outcome measure was determined by calculating the mean difference between groups in effect sizes with 95% confidence intervals and by calculating the response mean of all assessment measures across both groups (by dividing the mean difference of each variable at follow-up with the standard deviation of the mean difference). We also calculated the mean difference between groups in effect sizes with 95% confidence intervals at pre-test and follow-up for our potential outcome measures. In addition, we calculated the overall mean difference and standardised response mean based on the total sample in all potential outcome measures. Finally, we calculated the sample size that can be feasibly tested within a future definitive trial based on the mean difference between pre-test and follow-up on potential outcome measures and the rate of attrition we identified.

#### Acceptability of trial procedures

In order to determine the acceptability of oTTer trial procedures, both quantitative and qualitative data were collected.

We collected the following quantitative data:

- Number and proportion of families with quantitative data at each time point
- Missing item level data on intervention outcome measures at each time point

Questions in the interviews with both parents and LDWs considered the acceptability of various aspects of the trial and the assessment measures (see ‘Qualitative data collection and analysis’).

#### Protocol adherence and moderators

To track protocol adherence rates, the following data were collected:

- Frequency and number of sessions delivered and completion rate of families
- Length of time taken to train staff to deliver Talking Together
- Number of staff trained to deliver Talking Together
- Programme delivery quality assurance (collected routinely by BHT)

Potential moderators of attrition were also recorded using the qualitative data process described below (‘Qualitative data collection and analysis’).

#### Progression to trial criteria

Progression to trial criteria for recruitment, attrition, and protocol adherence was pre-defined based on Avery et al. [26]. These criteria were agreed on by multiple stakeholders [27] and used a traffic light system (‘red’ = indication not to progress to trial, ‘amber’ = potential progression to trial with consideration, and ‘green’ = progression to trial).

Progression to trial criteria for recruitment was measured by the number of families who were eligible for the trial (≥ 60% = green; 50–60% = amber; < 50% = red) and the number of families who consented to the trial (≥ 50% = green; 40–50% = amber; < 40% = red). The eligibility rate was calculated by dividing the number of eligible participants by the number of participants assessed for eligibility, excluding missing data. The consent rate was calculated by dividing the number of participants who consented by the number of participants who were eligible for the trial.

Progression to trial criteria for attrition were based on previous attrition rates from the Talking Together programme (2016–2017), using number of families that were retained from the original sample at the 6-month follow-up (≥ 80% = green, 70% = amber, and ≤ 70% = red).

Progression to trial criteria for protocol adherence was also assessed by calculating the proportion of participants seen within 4 weeks of each of the three assessment timepoints (Fig. 1; ≥ 80% = green, 60–80% = amber, and ≤ 60% = red).

#### Qualitative data collection and analysis

Qualitative data was collected using semi-structured interviews with LDWs and parents. Interviews lasted approximately 1 h and were conducted in parents’ homes and at BHT offices for the LDWs. Data were collected from 12 LDWs and 25 parents (13 intervention; 12 waiting control). Interviews were audio-recorded and transcribed, except for two parent interviews that were recording using interviewer notes rather than audio recordings due to participant preference or technical issues.

For the parent interviews, two researchers (KD and LT) independently coded two transcripts based on the research questions. The codes were discussed and refined, and a final coding framework was agreed upon. KD coded the remaining transcripts.

For the staff interviews, codes were initially identified by one researcher (KD) based on the research questions. A different researcher (LT) reviewed and assessed the codes over three transcripts to ensure they were suitable. Any comments or issues were discussed between the researchers, and the final coding framework was agreed upon. KD coded the remaining transcripts. The transcriptions and interviewer’s notes were analysed using NVivo 12 Pro.

## Results

### Participants

#### Recruitment and attrition

**Fig. 2:**
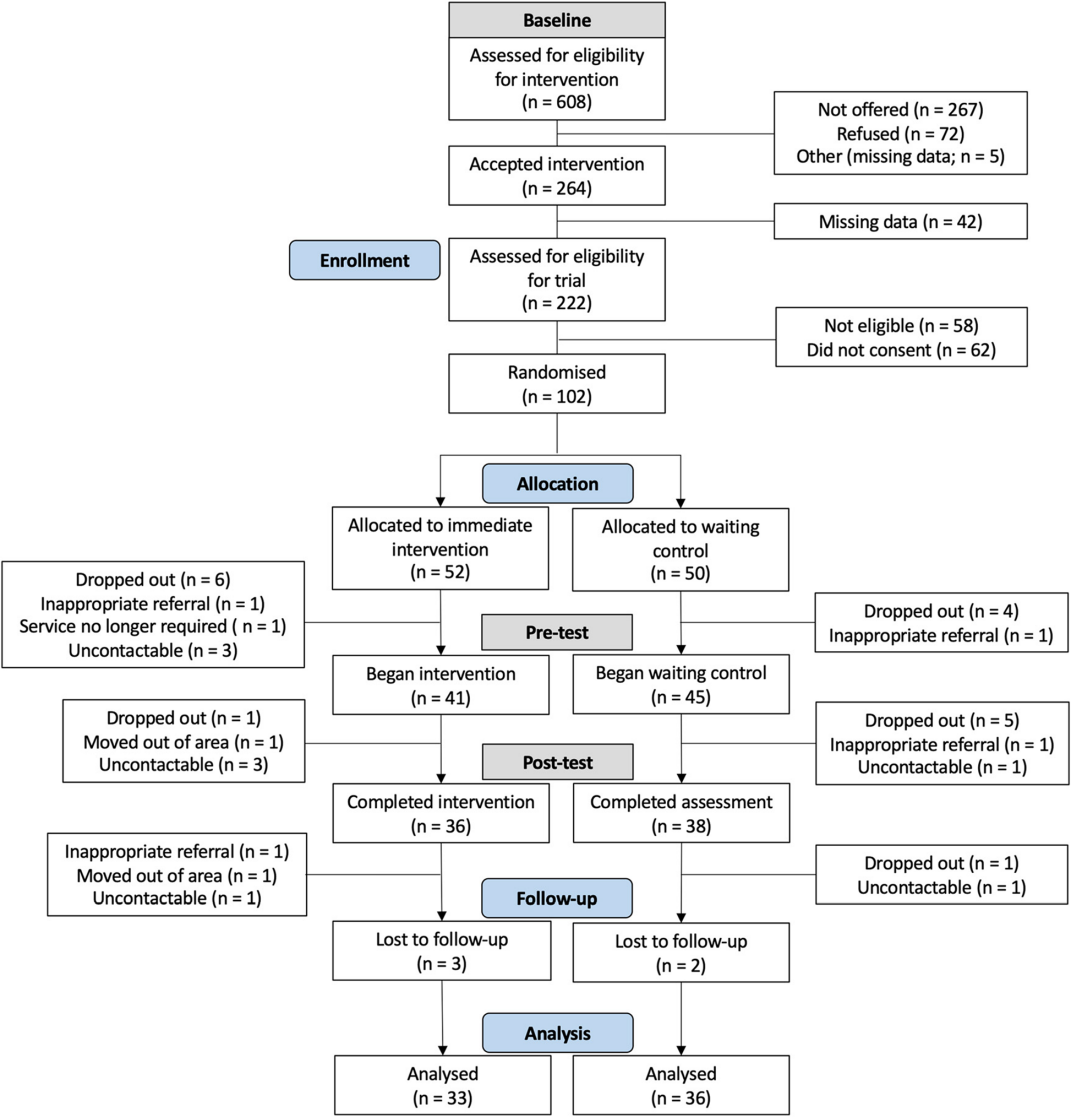
CONSORT diagram showing participant flow through the oTTer study

Figure 2 shows the CONSORT flow diagram for the oTTer study. Between 10 October 2018 and 14 June 2019, 608 families were assessed for eligibility for the intervention, of which 264 accepted the intervention. Of these, 42 families had missing data and the remaining 222 were assessed for eligibility for the trial. A total of 58 families were not eligible and 62 did not consent to the trial. Therefore, a total of 102 participants consented to the trial and went on to be randomised. Four eligibility violations were discovered during the trial (two due to participants speaking a language other than Urdu or Punjabi, which was not originally disclosed but became apparent during later assessments; two eligibility violations did not have reasons recorded). These four families were excluded from the trial but offered the intervention outside of it. Our final sample comprised 102 participants with children aged 2 to 2.5 years who were identified as being at risk of language delay and referred into Talking Together.

Randomisation resulted in approximately equivalent groups, with 52 participants allocated to the intervention arm and 50 to the waiting control. In the intervention arm, 41 participants (79%) began the intervention, 36 (69%) completed the intervention, and 33 (63%) were followed up. In the waiting control arm, 45 participants (90%) completed the pre-test assessment session, 38 (76%) completed the post-test assessment session, and 36 (72%) were followed up. Reasons for attrition are shown in Fig. 2.

**Fig. 3:**
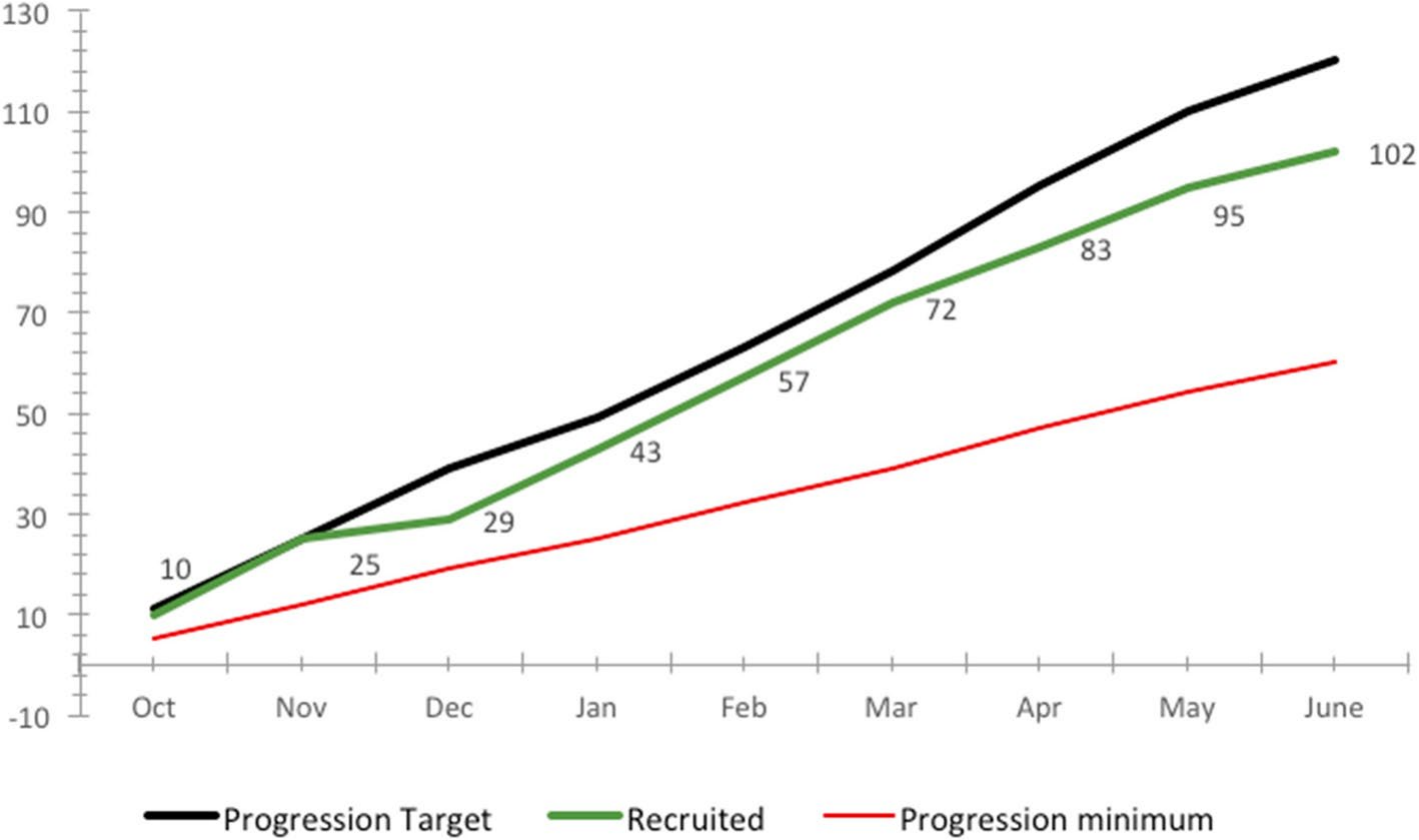
Recruitment across oTTer trial period: monthly figures required to meet the trial recruitment goal of 120 participants (black line) and the minimum recruitment figure of 60 participants (red line). Actual recruitment (participant n) is shown in green

Figure 3 shows how recruitment progressed during the recruitment period. Over the course of 9 months, recruitment was steady and showed no notable fluctuations.

#### Progression-to-trial criteria

Eligibility and consent rates were assessed as part of the progression criteria for the study, in line with the first objective of aim 1. The eligibility rate was 74% and the consent rate was 62%. Thus, the study met ‘green’ progression criteria for eligibility and consent rates.

The attrition rate at 6-month follow-up for the whole sample was 32%. This was higher than anticipated, and reasons for the attrition can be seen in Fig. 2. The progression criteria set out a minimum retention rate of 70%, putting the trial in ‘red’ for attrition criteria.

#### Demographics of oTTer participants as compared to the wider population

Demographics of the oTTer study participants are shown in Table 1. Participants in the trial were representative of the general Better Start Bradford population [12], answering the second objective of aim 1. Participants in the intervention and waiting control arms were similar in terms of children’s age, ethnicity, and home language, although there were more female participants in the waiting control group.

**Table 1:** Participant demographics for oTTer trial compared to non-oTTer trial participants in receipt of the Talking Together intervention

	Intervention	Waiting control	Total oTTer sample	Total non-oTTer families
N	52	50	102	162
Mean child age in months (SD)	25.87 (0.82)	25.8 (0.67)	25.83 (0.75)	26.51 (2.73)
*N* of females (%)	21 (40)	27 (54)	48 (47)	68 (42)
Child ethnicity (%)				
Asian/Asian British	38 (73)	38 (76)	76 (75)	107 (66)
White British	9 (17)	5 (10)	14 (14)	8 (5)
Other	5 (10)	7 (14)	12 (12)	47 (29)
Home language (%)				
English	31 (60)	28 (58)	59 (59)	52 (33)
Urdu	8 (15)	8 (17)	16 (16)	21 (13)
Punjabi	9 (17)	5 (10)	14 (14)	16 (10)
Other	^a^	^a^	11 (11)	68 (43)
Missing data	^a^	^a^	2	5

^a^Denotes data that was withheld due to small numbers to protect anonymity

#### Identification of potential outcome measures

An additional aim of this study was to pilot primary and secondary outcome measures for a full trial. This included assessing completion rates for all measures, as well as considering the performance of the intervention and control group on the measures. The standard deviations of the means for the primary outcome measure were also used to estimate the sample size required for a full-scale trial. Correlations between the language and non-language measures at pre-test and follow-up can be found in Supporting Information.

#### Reliability of potential outcome measures

Cronbach’s alphas (*α*) were computed for outcome measures with item level data based on scores at pre-test (with the exception of the ULA, which was only administered at baseline) to measure internal consistency (Table 2). All measures demonstrated acceptable levels of reliability in our population (*α* > 0.75), apart from the MORS Warmth measure, which showed poor reliability (*α* = 0.57).

**Table 2:** Internal reliabilities for potential outcome measure assessments

	Items	*N*	Cronbach’s alpha
Language screener (baseline)	10	95	0.77
HLE (pre-test)	16	82	0.76
MORS warmth (pre-test)	7	81	0.57
MORS invasive (pre-test)	7	83	0.78
SDQ total (pre-test)	10	82	0.76

Cronbach’s alphas could not be computed for two of the outcome measures (CDI, WellComm) because the data was not received at item level

*CDI* Oxford Communication Development Inventory-Short, *HLE* Home learning environment, *MORS* Maternal Object Relations Scale, *SDQ* Strengths and Difficulties Questionnaire

#### Responsiveness of potential outcome measures

**Fig. 4:**
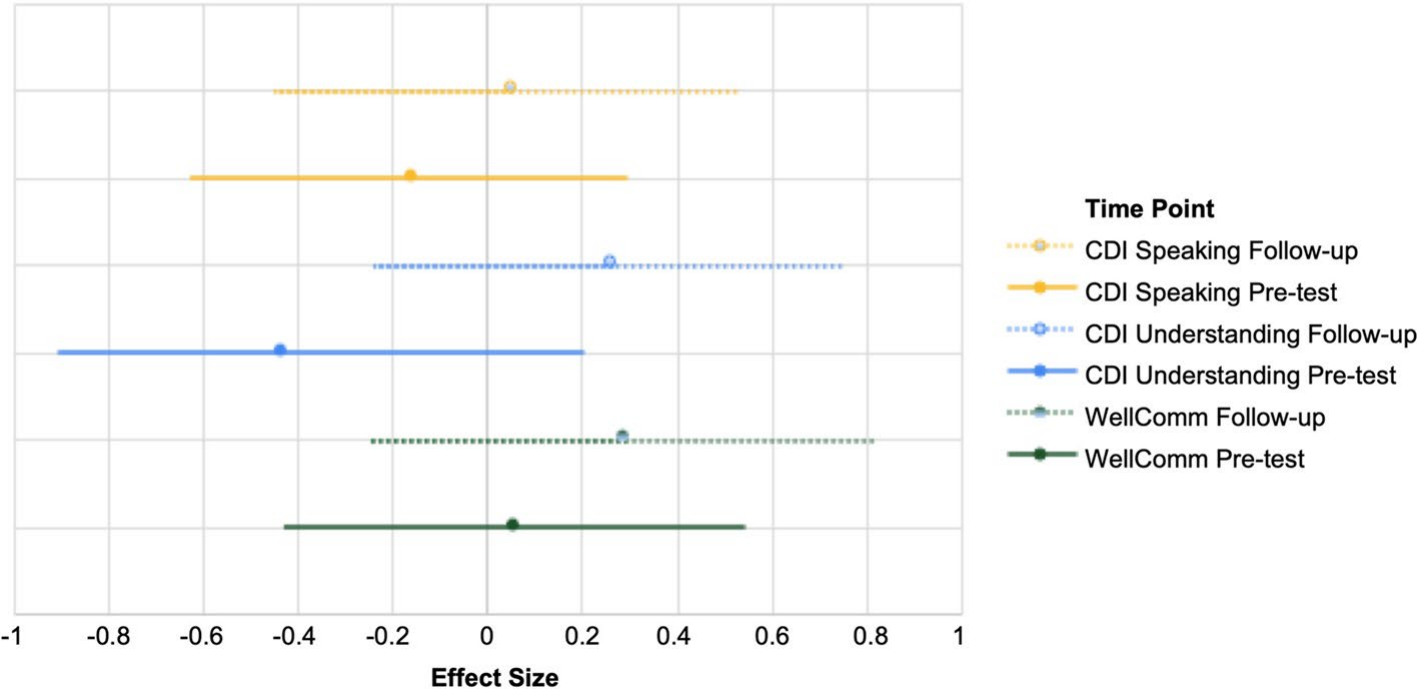
Effect size of mean difference between groups at pre-test and follow-up with 95% confidence intervals for language measures. Note: Lines of the same colour represent the same variable but at different time points. Solid lines represent pre-test, dotted lines represent follow-up. Effect sizes above zero represent an advantage for the intervention group

Descriptive statistics for all outcome measures are shown in Table 3. Effect sizes were calculated based on the mean difference between groups to identify trends in the primary and secondary outcomes. For the language measures (CDI, WellComm), mean differences between groups in effect sizes with 95% confidence intervals at both pre-test and follow-up are shown in Fig. 4. For all language measures, the follow-up results show a shift in the effect sizes to positive numbers, demonstrating an advantage for the intervention group. However, all measures at all time points had wide confidence intervals, suggesting considerable variability in the data across different participants. Despite this, for both the WellComm and the CDI Understanding, the effect sizes of *d* > *0.2* meet the threshold typically recognised as of educational significance [28], indicating a potential benefit of the intervention.

**Table 3:** Descriptive statistics for all potential outcome measures at all timepoints

	**Full oTTer sample N102**						**Group A intervention**						**Group B waiting control**					
	*N*	*M*	SD	Mdn	*R*	95% CI	*N*	*M*	SD	Mdn	*r*	95% CI	*n*	*M*	SD	Mdn	*r*	95% CI
**Language Screener (baseline)**	102	10.85	4.06	11.0	1–19	10.06, 11.64	52	10.48	3.96	10	2–18	9.40, 11.56	50	11.24	4.18	11.5	1–19	10.08, 12.40
**CDI Understanding**																		
Baseline	101	46.41	27.66	53.0	0–100	41.01, 51.80	52	44.40	27.71	49	0–100	36.87 51.94	49	48.53	27.74	55.0	0–86	40.76, 56.30
Pre-test	78	57.69	23.47	63.0	0–100	52.48, 62.90	33	51.85	26.58	54	0–91	42.78 60.92	45	61.98	20.14	66.0	18–100	56.09, 67.86
Post-test	71	67.99	19.43	72.0	0–98	63.47, 72.50	34	68.50	19.20	72	0–96	62.05 74.95	37	67.51	19.89	73.0	17–98	61.11, 73.92
Follow-up	65	78.66	15.86	81.0	28–99	74.81, 82.52	30	80.90	15.47	84	28–99	75.36 86.44	35	76.74	16.15	78.0	38–99	71.39, 82.09
**CDI Speaking**																		
Baseline	102	21.76	20.92	14.5	0–79	17.70, 25.83	52	19.71	21.23	11.5	0–79	13.9425.48	50	23.90	20.59	21.0	0–72	18.19, 29.61
Pre-test	78	26.78	22.70	18.5	0–82	21.74, 31.82	33	24.67	23.65	16	0–82	16.6032.74	45	28.33	22.12	25.0	1–74	21.87, 34.80
Post-test	71	38.51	25.70	40.0	0–96	32.53, 44.48	34	41.97	28.98	41	0–96	32.23 51.71	37	35.32	22.19	35.0	1–73	28.18, 42.47
Follow-up	65	58.89	28.61	64.0	0–99	51.94, 65.85	30	59.67	29.54	63.5	0–99	49.1070.24	35	58.23	28.20	67.0	2–96	48.89, 67.57
**WellComm**																		
Pre-test	70	2.14	0.8	2.00	1–4	1.95, 2.33	27	2.19	0.88	2.00	1–4	1.85, 2.52	42	2.12	0.76	2.00	1–4	1.89, 2.34
Post-test	59	2.47	0.75	3.00	1–4	2.28, 2.67	23	2.61	0.78	3.00	1–4	2.29, 2.93	36	2.39	0.73	2.00	1–4	2.15, 2.63
Follow-up	63	3.19	1.03	3.00	1–5	2.94, 3.44	29	3.31	1.00	3.00	1–5	2.95, 3.68	34	3.09	1.06	3.00	1–5	2.73, 3.44
**HLE Score**																		
Pre-test	83	29.60	10.55	31.0	1–49	27.33, 31.87	38	28.68	10.74	29.5	8–49	25.2732.10	45	30.38	10.45	32.0	1–49	27.32, 33.43
Post-test	71	31.04	10.28	31.0	9–54	28.65, 33.43	34	31.56	10.44	31	13–46	28.05, 35.07	37	30.57	10.26	30.0	9–54	27.26, 33.87
Follow-up	65	32.17	9.86	34.0	12–52	29.77, 34.57	30	34.13	8.80	36	12–49	30.99 37.28	35	30.49	10.53	31.0	12–52	27.00, 33.97
**MORS Warmth**																		
Pre-test	83	28.96	4.69	29.0	15–35	27.96, 29.97	38	28.84	4.47	29	17–35	27.42 30.26	45	29.07	4.91	30.0	15–35	27.63, 30.50
Post-test	70	30.26	4.44	31.5	13–35	29.22, 31.30	34	31.00	4.27	32	13–35	29.56 32.44	36	29.56	4.54	30.0	20–35	28.07, 31.04
Follow-up	65	30.68	4.00	32.0	18–35	29.70, 31.65	30	32.10	2.82	32.5	26–35	31.09 33.11	35	29.46	4.47	31.0	18–35	27.97, 30.94
**MORS Invasive**																		
Pre-test	83	11.04	6.19	10.0	1–32	9.71, 12.37	38	10.74	4.97	10.5	2–20	9.16, 12.32	45	11.29	7.10	10.0	1–32	9.21, 13.36
Post-test	70	9.97	5.41	10.0	0–24	8.70, 11.24	34	9.88	5.14	9.5	3–20	8.15, 11.61	36	10.06	5.72	10.0	0–24	8.19, 11.92
Follow-up	65	10.65	5.54	10.0	1–24	9.30, 11.99	30	9.33	4.14	9	1–21	7.85, 10.81	35	11.77	6.36	10.0	2–24	9.67, 13.88
**SDQ score**																		
Pre-test	82	8.13	4.07	8.0	1–18	7.25, 9.01	38	8.05	3.36	8	2–15	6.98, 9.12	44	8.20	4.63	8.0	1–18	6.84, 9.57
Post-test	71	7.23	4.36	7.0	0–20	6.21, 8.24	34	6.26	3.53	6	0–15	5.08, 7.45	37	8.11	4.89	8.0	1–20	6.53, 9.68
Follow-up	65	7.22	4.09	7.0	1–20	6.22, 8.21	30	5.90	2.88	6	1–11	4.87, 6.93	35	8.34	4.65	7.0	1–20	6.80, 9.88

*CDI* Oxford Communication Development Inventory-Short, *HLE* home learning environment, *MORS* Maternal Object Relations Scale, *SDQ* Strengths and Difficulties Questionnaire

**Fig. 5:**
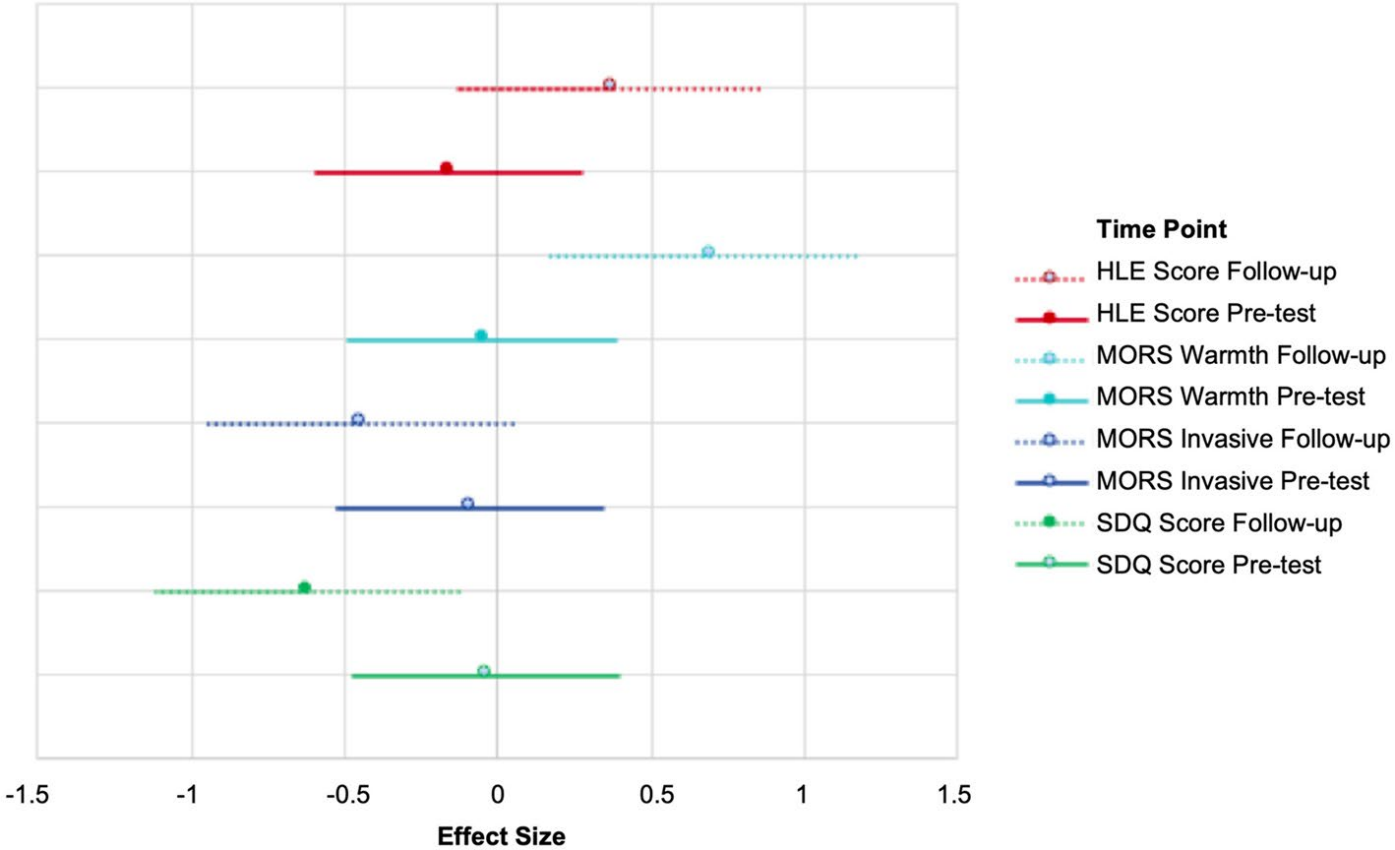
Effect size of mean difference between groups at pre-test and follow-up and 95% confidence intervals for non-language measures. Note. Lines of the same colour represent the same variable but at different time points. Solid lines represent pre-test, dotted lines represent follow-up. For the SDQ and MORS Invasive, effect sizes below zero represent an advantage for the intervention group. For MORS Warmth and HLE, effect sizes above zero represent an advantage for the intervention group

Similar analyses for the non-language measures are displayed in Fig. 5, which shows effect sizes with 95% confidence intervals. Results demonstrate a consistent trend for a shift in favour of the intervention group on all measures, suggesting a potentially beneficial impact of the intervention in improving children’s home learning environment, parental relationships with their child, and child emotional and behavioural difficulties.

We also calculated the standardised response mean for each measure in order to assess the responsiveness of the measures to change. There was a range in responsiveness of the measures from pre-test to follow-up (Table 4), with the most responsive language measure identified as the CDI (both Understanding and Speaking showed high responsivity). The most responsive non-language measure was the MORS Warmth.

**Table 4:** Mean difference between pre-test and follow-up on potential outcome measures in the total sample (intervention and control)

**Outcomes**	**Mean**	**SD**	**Standardised response mean**
CDI Understanding	33.95	28.20	1.20
CDI Speaking	36.51	30.89	1.18
WellComm	0.95	1.23	0.77
HLE	2.83	11.58	0.24
MORS Warmth	1.90	4.29	0.44
MORS Invasiveness	0.25	5.81	0.04
SDQ total	-0.90	4.56	-0.20

#### Estimation of feasible sample size for definitive trial

To address the fourth objective of aim 1, we calculated the feasible sample size for definitive trial by considered both the potential benefit of our chosen primary outcome measure (Oxford-CDI understanding) and the rate of attrition identified in oTTer (32%). Based on these parameters, a feasible sample size for a definitive trial would be approximately 735 with 80% power. However, if the rate of attrition was improved to match the usual attrition rates of Talking Together (27% attrition), a feasible sample size would be approximately 685 with 80% power.

#### Acceptability of trial procedures

##### Quantitative data

The completion rates for all outcome measures are reported in Table 5. Completion rates ranged from 68 to 100%, with a trend for poorer completion rates in the intervention compared to the waiting control group. The WellComm measure had the poorest completion rates, and this was particularly true for the intervention group.

**Table 5:** Assessment completion in raw numbers (and percentages) at all time points for each group

	**Baseline**		**Pre-test**		**Post-test**		**Follow-up**	
	**I**	**WC**	**I**	**WC**	**I**	**WC**	**I**	**WC**
ULS	52 (100)	50 (100)						
CDI	52 (100)	49 (98)	33 (80)	45 (100)	34 (94)	37 (97)	30 (91)	35 (97)
WellComm			27 (71)	43 (96)	23 (68)	36 (97)	29 (97)	34 (94)
HLEQ			38 (93)	45 (100)	34 (94)	37 (97)	30 (91)	35 (97)
MORS			38 (93)	45 (100)	34 (94)	36 (95)	30 (91)	35 (97)
SDQ			38 (93)	44 (98)	34 (94)	37 (97)	30 (91)	35 (97)

*I* Intervention group, *WC* Waiting control group

##### Qualitative data

Qualitative data was used to consider the acceptability of trial procedures to address the fifth objective of aim 1.

##### Potential outcome measures

Practitioner and family feedback indicated that although a few parents found the CDI difficult to complete and disheartening, it was the easiest to administer of the language measures in the home setting. The WellComm was unacceptably time-intensive for LDWs and as a result was not consistently completed. This may be due to administering the WellComm in a busy home setting, as opposed to a structured early years setting as it was intended to be used. LDWs indicated that some of the non-language measures were initially awkward to ask about, specifically those that asked about challenging behaviour, but families themselves did not report concerns about this. Across all measures, practitioners noted that concerns were remedied by raising them with the wider team, who aided time management issues around workload or provided necessary support.

##### Trial barriers

The qualitative analysis identified several reservations about the trial:

1. Poor understanding of the distinction between the trial and access to the intervention: both practitioners and parents struggled with the distinction between the trial to assess the intervention, and the availability of Talking Together itself. This was supported by practitioner reports that families withdrew once contacted to arrange for the pre-test visit with further explanation about the study, and by the rate of attrition, which was highest between baseline and pre-test (16%). Practitioners felt it was challenging to explain the trial to parents in a way they understood, particularly when explaining randomisation, and about their right to receive the intervention without taking part in research. Some, but not all, of this was due to language barriers and literacy difficulties.
2. High number of potential outcome measures: The qualitative analysis revealed that the number of measures carried out by LDWs was difficult for both families and the practitioners, and may have contributed to attrition. Practitioners felt that the assessment measures increased their workload and limited their time with the target families. The large amount of data entry because of the number of outcome measures was also a concern.
3. Use of a waiting control group: Practitioners and some parents felt that the 6-month wait for the waiting control group was too long. Some practitioners did not refer all eligible children into the trial to avoid the ‘risk’ of them being allocated to the waiting control group. Some parents reported that the potential to be allocated to a waiting control group discouraged them from taking part in the trial. This data directly addresses the first objective of aim 1 and suggests that while possible, a waiting list control design is not without issue in this population.
4. Confusion regarding eligibility criteria: Some practitioners and families found the eligibility criteria confusing, resulting in four violations of eligibility during the trial (see ‘Recruitment and attrition’).
5. Difficulty scheduling data collection at follow-up within trial team: Some practitioners reported that it was difficult to schedule the follow-up sessions, as these required both the research assistances and the practitioners to be available.

##### Trial facilitators

There were four prominent facilitators of the trial identified through the semi-structured interviews:

1. *Home-visiting nature of the programme:* Both practitioners and families appreciated that the trial was delivered in the home. Parents highlighted the advantage of not needing to travel in order to participate.
2. *Good rapport between practitioners and families:* LDWs and families highlighted the positive effect of developing a rapport during the course of the trial and the intervention. Parents reported feeling comfortable with practitioners, and that their children enjoyed the visits.
3. *Presence of two practitioners during assessments*: LDWs appreciated visiting families in pairs, as this allowed one to focus on data collection, whilst the other practitioner engaged with the child or family.
4. *Good support within the data collection and intervention team:* LDWs felt well supported in their team throughout the trial and able to raise concerns, which helped mitigate some of the barriers identified, such as workload.

#### Protocol adherence and moderators

##### Quality assurance

Due to an error in data storage, it was not possible to formally monitor programme delivery quality assurance; however, as this is routinely monitored by the service provider BHT, fidelity was likely sufficient, despite the lack of research data available.

##### Potential moderators of attrition

The following moderators of attrition were identified following the qualitative analysis:

1. *Provision of language packs for waiting control group:* LDWs reported that the language packs were a useful incentive for the waiting control group.
2. *Reforming consent procedures:* Attrition could be improved by reforming consent procedures, such as by providing simple language explanations supported by clear pictorial infographics at recruitment.
3. *Improved training for LDWs regarding eligibility:* The qualitative analyses identified that peer support within the team was crucial to overcoming concerns around allocation to the waiting control group and that some found the eligibility criteria confusing. Improved training to improve confidence around eligibility with built in peer support is thus indicated for a full trial.
4. *Reduced number of outcome measures*: One aim of the feasibility study was to identify potential outcome measures to measure effectiveness of the intervention. As our analyses revealed that the Oxford-CDI and the MORS were the most suitable in terms of both administration and responsiveness to the intervention, only these would be administered for a full trial, which would aim to improve attrition rates by reducing burden on families and increasing time spent with LDWs.

##### Progression-to-trial criteria

An important component of the first object of aim 1 was understanding whether assessments were conducted as planned. Across the groups and time points, adherence to prespecified assessment timepoints ranged from 51 to 89%, with poorer adherence in the intervention group, and the poorest adherence for both groups at pre-test (Table 6). On average, adherence to assessment timepoints was 66%, placing protocol adherence in ‘amber’ progression criteria.

**Table 6:** Waiting time in weeks for participants between each assessment timepoint

	Intervention			Waiting control		
	Baseline to pre-test	Pre-test to post-test	Post-test to follow-up	Baseline to pre-test	Pre-test to post-test	Post-test to follow-up
Mean (SD)	5.72 (6.54)	8.07 (2.79)	28.32 (3.96)	4.18 (1.87)	6.95 (1.28)	27.29 (4.51)
Range	1.86–43.86	4.86–15.86	22.00–39.29	2.00–11.29	5.00–10.00	22.00–50.00
% meeting target	51	67	58	64	89	69

#### Time and resources for Talking Together

To address the second objective of aim 2, we considered the training requirements of delivering the intervention. The time taken for one LDW to attend training for Talking Together was 31.5 days, and delivery of Talking Together to one family was 1.5 days. Training costs were relatively high upfront per LDW (£6768), but would only apply to LDWs who were new to the service, and also need to take into account that one LDW would deliver the intervention to multiple families. Therefore, calculated on a basis of one LDW delivering Talking Together to ten families (£676.80) and the cost of delivery per family (£153.50), this equates to a unit cost of £830.30. This compares favourably to other home-visiting programmes—for example, ParentChild + and Parents as First Teachers equate to a unit cost between £1,000 and £2,000 [29]. A full breakdown of costs can be found in the Supporting Information (Tables S1 and S2).

## Discussion

This study assessed the feasibility of conducting a full-scale trial of the home-visiting intervention Talking Together, which aims to improve child language skills through parental education. Findings suggested that the intervention was well received. The trial had good levels of recruitment, even with a waiting control group, and provided extensive information to inform and optimise a future full-scale trial. Based on these results, this study met its objective and found that it would be possible and justified to conduct a full-scale randomised controlled trial, albeit with some modifications to improve recruitment and retention.

Recruitment, protocol adherence, and attrition were identified as key criteria for determining the feasibility of progression onto a full trial. The results suggested that recruitment was an area of strength, placing it within ‘green’ for progression criterion. Whilst these results were encouraging, the qualitative data revealed that practitioners were not always certain regarding eligibility criteria, and this resulted in four eligibility violations. Additionally, individual practitioners who were concerned about the 6-month wait for the control group did not offer the study to all eligible participants. This may have caused a selection bias and suggests future work would need to include greater oversight of the screening process, as well as additional training for practitioners.

Attrition at follow-up was higher than expected and was considered as ‘red’ according to the progression criteria. However, it is worth noting that attrition from intervention programmes running within the community (as part of the Better Start Bradford programme) range from 14 to 60%, with an average of 36% (from internal data within the Better Start Bradford Innovation Hub). As such, retention is a known challenge in this community. Furthermore, attrition from the intervention by participants receiving standard practice was 27%, whilst for participants of the trial, it was 32%, suggesting only a slight increase in attrition under trial conditions. Future trial numbers were calculated to account for anticipated attrition and any future trial would need to improve attrition rates using the information gleaned from the qualitative analysis.

Protocol adherence, which measured whether participants were seen for assessments within the timeframes set out in the protocol, had an average progression criterion of ‘amber’. There was particularly poor adherence at pre-test and follow-up and in the intervention group. This group difference most likely reflects the greater flexibility in the control group assessors, as the waiting control assessments were carried out by research assistants with no treatment caseload. It would be vital in a future trial to consider how to ensure both groups are seen within the appropriate timeframe by increasing capacity in the teams and streamlining administrative processes associated with recruitment.

Another key objective (aim 1) of this study was to identify appropriate primary and secondary outcome measures to take forward in future studies. Practitioners found the number of assessments and paperwork in sessions and the accompanying data entry burdensome. However, the identification of key primary and secondary outcome measures from the feasibility study would allow for a condensed assessment battery in future. Results from both the quantitative and qualitative analyses suggested that the primary outcome measure should be the Oxford CDI Understanding subscale, as this showed good levels of completion and strong reliability. The Oxford CDI Understanding also showed high levels of responsiveness, suggesting it would identify changes over time, allowing for an evaluation of the effectiveness of the programme. Although the WellComm had the advantage of being a holistic assessment of language as opposed to vocabulary only, it was not consistently administered, and practitioners considered it more time-consuming than the CDI. Data from the MORS Warmth measure indicated that this would be a suitable secondary outcome measure, as it had strong levels of completion and acceptable levels of responsiveness.

Interviews with the LDWs and parents revealed important lessons for a future trial. LDWs and parents both noted the value of having a home-based programme, as it was more convenient for families and allowed for practitioners to gain a greater understanding of the home learning environment of the children. Both groups were also positive about the relationships that parents and children were able to develop with the LDWs, and this was considered a real strength of the programme. However, LDWs were concerned that parents did not fully understand the study at the point of consent, and the parent interviews revealed a lack of knowledge about the trial and participants’ ability to receive the intervention without participating in the study. It would be vital in future to ensure that the consent process was improved so that participants were fully informed. The 6-month waiting period was also a concern for practitioners, and qualitative data showed that it was the primary reason eligible participants refused consent to the study. A future trial would need to invest more time in developing resources with parents to optimise understanding of the recruitment and randomisation approach. This may include the use of different media to facilitate decisions, such as infographics that can be accessed without practitioners and without reliance on adequate literacy levels. Parents and carers may also need more time to consider participation in the trial.

A nurturing home learning environment has been even more paramount in recent years, given the multiple lockdowns that families have endured as a result of the COVID-19 pandemic. Within this context, early years settings closed to all children apart from those of key workers and vulnerable children, hence, families were required to keep their children at home with many also juggling work commitments and health concerns. Some reports have suggested that these lockdowns have exacerbated existing inequalities [30]. As a result, identifying evidence-based early interventions that can improve children’s language outcomes is imperative for the future.

## Conclusions

The oTTer study has determined that the Talking Together intervention is well received by its community. Key potential outcome measures were also identified as acceptable and responsive, and processes for improving the consent process and attrition rates, as well as a need for more intensive training of practitioners, were identified. A full-scale trial of the programme would thus be feasible with the improvements identified from this feasibility study.

## Supplementary Information

**Additional file 1: Table S1.** Estimated training costs for one LDW to deliver Talking Together. **Table S2.** Estimated delivery costs for Talking Together for one family.

## Data Availability

Data from the feasibility trial will be available after final evaluation and publications of the study and can be requested following procedures described on the Born in Bradford website: www.borninbradford.nhs.uk.

## Abbreviations

CDI: Communicative Development Inventory
HLEQ: Home Learning Environment Questionnaire
LDWs: Language development workers
MORS: Maternal Object Relations Scale
SDQ: Strengths and Difficulties Questionnaire
